# The Influence of Secondhand Smoke Exposure on Birth Outcomes in Jordan

**DOI:** 10.3390/ijerph7020616

**Published:** 2010-02-22

**Authors:** Nesrin N. Abu-Baker, Linda Haddad, Christine Savage

**Affiliations:** 1 School of Nursing, Jordan University of Science & Technology, P.O Box 3030, Irbid 22110, Jordan; E-Mail: nesrin@just.edu.jo; 2 Department of Family and Community Health & Institute for Drug and Alcohol Studies, School of Nursing, Virginia Commonwealth University, Richmond, P.O. Box 980567, VA 23298-0567, USA; 3 College of Nursing, Department of Public Health Science, College of Medicine, University of Cincinnati Academic Health Center, P.O. Box 210038Cincinnati, OH 45221-0038, USA; E-Mail: savagece@ucmail.uc.edu

**Keywords:** Secondhand Smoke (SHS), environmental tobacco smoke, Jordan, birth outcomes, neonates weight

## Abstract

This study investigates how secondhand smoke (SHS) exposure influences neonatal birth weight in Jordan, a country with high smoking prevalence. The findings revealed that as the average number of SHS exposure hours per week increased in the second trimester, the neonatal birth weight decreased while holding all covariates constant. Women who reported a higher average number of SHS exposure hours per week from work in the second trimester, home in the third trimester, and outside in the third trimester were at greater risk for having a low birth weight neonate than women who reported a lower average number of SHS exposure hours.

## 1. Introduction

Evidence is emerging that secondhand smoke (SHS) exposure during pregnancy increases the risk of low birth weight among the infants of non-smoking women. This may be of particular importance in Jordan due to the high prevalence of SHS exposure experienced by non smoking women. Although SHS is a known cause of premature death and disease among women and children [1], most studies examining the relationship between SHS exposure in women and children have been conducted in developed countries where exposure is relatively low and with patients for whom maternal smoking during pregnancy is relatively high [2–5]. In contrast, effects of SHS exposure (including product type, magnitude, and time of exposure) among women and children in developing countries has not been well studied even though smoking prevalence is much higher in those countries because tobacco control initiatives are minimal [6]. The lack of data about the impact of SHS exposure on women’s and children’s health outcomes in developing countries with a high smoking prevalence among the general population and low maternal smoking prevalence during pregnancy makes it difficult for researchers, lawmakers, health professionals, and families to make informed decisions about tobacco prevention. Globally, about one-sixth of all newborns are low birth weight (LBW) (LBW, <2,500 grams), which is the single most important underlying risk factor for neonatal deaths [7,8]. In undeveloped countries, including Jordon, only about half of the newborns are weighed at birth, and gestational age is known for a smaller proportion of those newborns. Moreover, LBW babies who survive the critical neonatal period may suffer long term impaired physical and mental growth. Therefore, an early identification and prompt referral of LBW newborns is vital in preventing neonatal deaths and long term physical and mental impairment.

### 1.2. Hypotheses

In this prospective survey study, the influence of SHS exposure on neonatal birth weight and the risk of having a LBW neonate as a result of Jordanian non-smoking women pregnant women being exposed to SHS were investigated. The research hypotheses were:

Among non-smoking Jordanian women giving birth at a major medical center in northern Jordan:

1. Increasing the reported average number of SHS exposure hours per week from home, work, and outside in the first, second, and third trimesters lead to decreasing the neonatal birth weight significantly after controlling for covariates.
2. Women who reported higher average number of SHS exposure hours per week from home, work, and outside in the first, second, and third trimesters were at significantly greater risk for having a LBW neonate than women who reported lower average number of SHS exposure hours after controlling for covariates.

### 1.3. Health and Smoking Behavior in Jordan

Jordan is a developing, low-to-middle income country that is advancing and adapting to a twenty-first century economy. It is geographically situated between Saudi Arabia, Iraq, Israel, and Syria and thus is directly embedded in the culture, societal trends, and concerns of the Middle East as a whole. Administratively, Jordan has three regions with 12 governorates. The gross domestic product is $8.9 billion, which yields a mean of $1,744 per capita [9–11].

Jordanians view smoking as a social habit; having coffee and cigarettes with friends and family members is deeply rooted in the culture. From 2002 to 2006, 50% of adult men and 11% of adult women smoked [12–14]. A recent series of surveys in Jordan estimated smoking rates of 24.9% among youth with a 76.3% self-report of SHS exposure [15], 22.4% among male physicians [16], and 28.6% among college students [17,18], which rose to 35% in 2008 [19]. Furthermore, there are no restrictive antismoking regulations in the workplace. Generally, women do not encourage their husbands or visitors to smoke outside their homes or in a different room.

Jordan and many other Middle Eastern countries lack systematic knowledge and surveillance data about the harm and economic effects of smoking and second-hand smoke exposure. Unfortunately, many aspects and social attitudes of using tobacco are shaped by their acceptance in Jordanian Arab culture [20]. Therefore, this study concerning the magnitude of exposure and the harmful effects of SHS on neonate and newborn weight may provide impetus for Jordanian society and policy makers to develop guidelines to reduce SHS exposure.

### 1.4. Theoretical Framework

**Figure 1.**
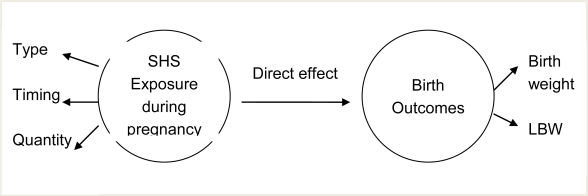
Study framework.

Few reports chronicling the possible influence of SHS exposure among non-smoking Jordanian pregnant women on birth weight in this population were found. Further, LBW neonates have a greater risk for mortality and morbidity in the first week of life [21]. Therefore, it is imperative to further examine the dangers associated with this potential environmental risk. This is especially important in countries, such as Jordan, where there is a high prevalence of smoking in the male population [22–24]. Moreover, most researchers have focused on birth weight and SHS exposure during pregnancy in general. Further knowledge is needed about the type of SHS exposure (at home, work, or outside home/work), the quantity of SHS exposure (average number of exposure hours per week), and the timing of SHS exposure (first, second, and third trimesters). Two major variables are involved in the relationship between SHS exposure during pregnancy and birth outcome. (See Figure 1.) These variables are: SHS exposure during pregnancy, and birth outcome.

*Secondhand smoke exposure*. Pregnant women are usually exposed to SHS in different places for different durations and at different times during pregnancy. For the purpose of this study, the assessment of SHS exposure included three major components: the type of SHS exposure (at home, work, or outside), the quantity of SHS exposure (average number of exposure hours per week), and the timing of the SHS exposure (first, second, and third trimesters).

*Direct effect of SHS exposure.* The toxins in SHS directly cause harmful effects in the fetus [25–28]. Nicotine is known to be vasoactive and is thought to reduce fetal circulation via the placenta [29–34]. Cotinine, a major metabolite of nicotine, has been measured in follicular fluid and amniotic fluid. Carbon monoxide is known to deplete fetal oxygen supplies [23,35–37].

*Birth outcome*. For the purpose of this study, birth outcomes included low birth weight and actual birth weight. Birth weight was defined as the number of grams that represented the neonate’s weight at birth. Low birth weight was defined as a birth weight of less than 2,500 grams

### 1.5. Potential Covariates of Newborn Weight

The rate of newborn, infant, and early childhood growth is a sensitive indicator on the impact of SHS exposure. Multiple maternal and neonatal factors are also associated with birth weight. Two separate secondary data analyses of survey data for 35 developing countries showed that maternal education and household wealth have a strong positive association with height for age and early life diseases, with substantial shared variance between these two variables in explaining child height for age [38,39]. Clearly, factors such as parental stature and gestational age have significant influences on birth weight. The incidence of LBW in neonates is significantly higher for first pregnancies, high maternal weight gain during pregnancy, maternal age less than ≤18 or ≥35 years, maternal alcohol or drug use, and for female neonates [38,40–44].

## 2. Methods

### 2.1. Setting

Princess Badia Teaching Hospital in Irbid, northern region of Jordan served as the setting for recruiting study participants for this study. This hospital is part of the Ministry of health (MOH) Public health sector hospitals in Jordan and is the main public hospital that specializes in obstetric and gynecology in the northern region of Jordan. About 900 deliveries occur in this hospital every month. In Jordan, 95% of the deliveries occur in hospitals. 65% of these deliveries occur in public MOH hospitals [22].

### 2.2. Sampling

Given a power analysis using a moderate effect size of 0.50 and a priori power estimation of 0.80, for a two-tailed test with alpha previously set at 0.05, at least 67 subjects for each group are required to have confidence in the findings [45]. Birth weight was the primary outcome of this study. A minimum of 100 participants were needed to study the influence of SHS exposure on birth weight. Since LBW was the second birth outcome and the LBW rate in Jordan is 10.6%, a larger sample was selected to have adequate number of participants with LBW. The total sample size was 300 participants with a 2% refusal rate to participate.

Systematic sampling was used for this study. On each day that was designated for data collection, the current patient list on the post delivery floor was reviewed. Every third patient was selected as a potential participant prior to knowing about all the inclusion criteria. Participants met eligibility requirements if they were 18 years of age or older at the time of recruitment, reported that they did not smoke tobacco for one year prior to conception, their pregnancies resulted in a single live birth, and their pregnancies resulted in full term births (equal to or more than 37 weeks of gestation). The eligibility criteria of having a non-malformed full term birth were also verified from the medical records. Women who were not aware of having malformed or preterm births were excluded after they were asked to fill out the SHS exposure questionnaire.

Mothers who had medical problems diagnosed before pregnancy (diabetes, hypertension, heart problems, lung problems, and anemia), or medical problems diagnosed during pregnancy (gestational diabetes, gestational hypertension, and anemia) were excluded from the study.

### 2.3. Measurement

#### Environmental tobacco smoke exposure questionnaire

The Environmental Tobacco Smoke Exposure questionnaire was used to measure SHS exposure from home, work, and outside in the first, second, and third trimesters. The questions in the SHS questionnaire were adapted from the Pregnancy Risk Assessment Monitoring System (PRAMS) questionnaire [46]. The PRAMS questionnaire includes questions about facts such as age, height, and education. It also includes self-reported maternal habits during pregnancy, including tobacco use and SHS exposure. No information on reliability and validity was found for this questionnaire. Since the study was conducted in Jordan, where people speak Arabic, the SHS questionnaire was translated into Arabic by the investigators who are fluent in both Arabic and English. Subsequently, the Arabic copy was translated back into English by another investigator who is also fluent in both languages.

Environmental tobacco smoke exposure at home was defined as the average number of hours per week in which the pregnant women was exposed to SHS from her husband or anyone else inside her house. This information combined with the mother’s report of her time spent in the home was used to help quantify the predictor variable, the average of hours per day the woman was exposed to cigarette smoking by her husband or anyone else inside her house in each trimester.

Environmental tobacco smoke exposure at work was defined as the average number of hours per week in which the pregnant woman was exposed to SHS at her job. Environmental tobacco smoke exposure at outside places was defined as the average number of hours per week in which the pregnant woman was exposed to SHS from outside home or work, such as relatives’ homes, restaurants, cars, and doctors’ offices.

The SHS exposure questionnaire was also used to measure potential covariates. The potential covariates included neonate’s characteristics, maternal demographic data, maternal obstetric history, and current delivery. The neonate’s characteristics included gender and gestational age. Maternal demographic data included mother’s age, height, total family income, education, and occupation during pregnancy. Maternal obstetric history and current delivery included parity, weight before pregnancy, weight gain during pregnancy, initiation of prenatal care, alcohol use during pregnancy, and illicit drug use during pregnancy.

#### Preliminary study

The Arabic version of the SHS Exposure Questionnaire was pilot tested on a group of Jordanian women (n = 30) in order to test data collection procedures and compute the reliability coefficient and construct validity of the Arabic version. The total scale yielded a Cronbach’s alpha of 0.78. A factor analysis using principle components extraction was performed to ascertain whether the Arabic version of the SHS exposure questionnaire items would load in a pattern similar to the one in the English data sets. The pilot study revealed three factors which matched with the original English questionnaire. Together the three factors explained 98.9% of the total SHS exposure variance.

Finally, based on the feedback from participants, the last three items of the questionnaire were modified to include outside places of exposure such as relative homes in addition to public places such as restaurants, cars, and doctors’ offices.

#### Medical records

Medical records were also used to validate self-reported data related to exclusion criteria, including medical problems diagnosed before pregnancy (diabetes, hypertension, heart problems, lung problems, and anemia) and medical problems diagnosed during pregnancy (gestational diabetes, gestational hypertension, and anemia). Anemia was defined as hemoglobin concentration of less than 11 gm/dL in the first and third trimester or hemoglobin concentration of less than 10.5 gm/dL in the second trimester [28]. Data requested from maternal records also included prescription drugs during pregnancy, if there were any.

Birth weight was defined as the number of grams that represented neonate’s weight at birth as recorded in the neonate’s medical records. Low birth weight was defined as a neonate with less than 2,500 grams at birth as recorded in the neonate’s medical records. The actual birth weight in grams was obtained from the medical records. Based on birth weight, neonates were classified as LBW or normal birth weight neonates.

Finally, data were collected from medical records to measure potential neonatal covariates. These covariates included neonate’s gender (if the neonate is a male or a female) and gestational age (number of pregnancy weeks since first date of mother’s last menstrual period). Data requested from the neonate’s record also included the date of delivery and the method of delivery.

The committee for protection of human subjects at Jordan University of Science and Technology approved the study design and consenting methods prior to beginning the study. Also, formal permission to conduct this study in Jordan was obtained from the director of Princess Badia Teaching Hospital. A brief description of the study purpose was given to participants before the questionnaire was distributed and voluntary consent was obtained. Participants were informed that their responses would be kept confidential and that all results would be presented as an aggregate data. Participants were asked for their permission to access some information from their medical records and their neonates’ medical records.

The unit of measurement for initiation of prenatal care was the month of pregnancy and that in the multiple regressions analysis it was quantified by months. The mother’s occupation during pregnancy was quantified as; the mother was asked if she have a job outside home (yes, no), if yes, how many weeks did she work in each trimester, how many hours did she work on average per week, While pregnant, if she had regularly been in the same room with someone who smokes cigarettes, and finally on average how many hours per week she has been in the same room with someone who smokes cigarettes at her job in the 1st, 2nd, and 3rd trimester. The last variable was entered in the regression analysis as a predictor of SHS exposure at work.

The covariates included in the analysis were neonate’s gender, gestational age, number of mother’s previous pregnancies, weight gain during pregnancy, initiation of prenatal care, maternal age, maternal weight before pregnancy, mother’s years of education, mother’s occupation during pregnancy, and total family income. Mean number of living children included the neonate and all other living, biological children of the participant.

## 3. Results

### 3.1. Maternal and Neonate’s Characteristics

*The mean age of the study participants was 28 years (SD = 5.52), the mean height* was 160.2 centimeters (SD = 6.25), the mean years of education was 11.4 years (SD = 2.79), and the mean total family income was 2,888 $US per month (SD = 105.22). 42.3% of the study participants reported having an education level of less than high school; 33.3% completed high school education, and 24.3% completed post high school education. With regard to the father’s education level, 45% reported having an education level of less than high school; 32.3% completed high school education, and 22.6% completed post high school education. Only 10% of the study participants reported having jobs outside home (*n = 31*). Many of the demographics fit the national profile as the mean height for women is 160 Centimeters, and 55% of women have more than high school education. However the reported total family income was lower than the national average ($ 4,820) [10].

### 3.2. Maternal Obstetric History and Current Delivery

The mean weight before pregnancy of the study participants was 63 kilograms (SD = 10.53); the mean weight gain during pregnancy was 11.1 kilograms (SD = 3.48); the mean number of pregnancies was 3.5 pregnancies (SD = 2.24); and the mean number of living children prior to the subject neonate was three (SD = 1.83. 79% of the participants reported initiation of prenatal care in the first trimester; 20% reported initiation in the second trimester; and 1% reported initiation in the third trimester (see Table 1).

The mean birth weight of the participants’ neonates was 3,245.95 grams (SD = 444.06), and the mean gestational age was 39.3 weeks (SD = 1.19). Furthermore, 50.3% of the participants had baby boys (*n* = 151); 76% had vaginal deliveries (*n* = 228); 24% had caesarean sections (*n* = 72); and 9.3% had LBW neonates (*n* = 28).

### 3.3. Environmental Tobacco Smoke Exposure

The type of SHS exposures in this study were classified as exposure from all places, exposure from two places, exposure from one place, or no exposure. Two percent of the participants reported SHS exposure from all places; 55% reported SHS exposure from home and outside; 30.3% reported SHS exposure from outside; 7.7% reported SHS exposure from home; and 2.7% reported no SHS exposure (Table 2).

The mean SHS exposure hours from the home environment were the highest across all the three trimesters. They were 13.1, 13.2, and 13.1 hours per week in the first, second, and third trimesters respectively. The mean SHS exposure hours from work were 5.0, 4.8, and 4.5 hours per week in the first, second, and third trimesters respectively. The mean SHS exposure hours from outside were 4.8, 4.85, and 4.85 hours per week in the first, second, and third trimesters respectively (Table 3).

With regard to SHS exposure at home, 65.3% reported SHS exposure at home. Among subjects who reported SHS exposure at home, 52.7% reported SHS exposure from their husbands only, 10% from their husbands and other smokers at home, and 2.7% from smokers other than their husbands. (The 2.7% had husbands were nonsmokers.) Furthermore, the average reported number of cigarettes that were smoked by the exposed mother’s husband in the home was 16.3 cigarettes per day (SD = 15.89).

### 3.4. Comparison between the Groups of Participants Who Had Normal Birth Weight Neonates and who had LBW Neonates

Before running the multiple regression and logistic regression, an equivalent analysis was conducted between the group of participants who had normal birth weight neonates (*n* = 272) and the group of participants who had LBW neonates (*n* = 28) in terms of potential covariates. Descriptive statistics, t-tests for continuous variables, and chi-square tests for categorical variables were performed to compare the two groups. There were 272 participants who had normal birth weight neonates (90.7%) and 28 participants who had LBW neonates (9.3%). This is similar to the LBW rate in Jordan as a whole (10.6%) [47]. The mean birth weight for the first group was 3338.8 grams (SD = 351.5), and the mean birth weight for the second group was 2343.6 (SD = 110.9).

Table 4 shows the participants’ weight before pregnancy *t* (298) = 3.2, p < 0.001, weight gain during pregnancy *t* (298) = 2.1, p = 0.036, and gestational age of the neonate *t* (298) = 2.8, p < 0.005 were significant covariates (p < 0.05) (Table 4). These covariates were included in the final logistic regression model because they were significantly different between the two groups.

There was no significant difference between the two groups in terms of neonates’ gender χ^2^ (1, *n=* 300) = 0.69, p = 0.41 (two-tailed), mother having a job outside home χ^2^ (1, *n =* 300) = 1.89, p = 0.17 (two-tailed), or initiation of prenatal care in the first trimester χ^2^ (1, *n =* 300) = 1.87, p = 0.17 (two-tailed). There were no participants who used drugs or alcohol during pregnancy.

### 3.5. The Influence of SHS on Neonatal Birth Weight

A stepwise multiple regression analysis was conducted to test the first hypothesis, where the dependent variable, which is the neonatal birth weight, could be explained by the independent variable, which is the reported average number of SHS exposure hours per week, in the first, second, and third trimesters. Multicollinearity was examined between the independent variables before the stepwise multiple regression analysis, to test for collinearity. Specifically the highest correlation coefficient between any independent predictor variables was only 0.49. The variance inflation for the independent variables was calculated with a result of 0.91, which indicated a high level of tolerance thus low collinearity.

The potential covariates that were entered in the model included the neonate’s gender, gestational age, number of mother’s previous pregnancies, weight gain during pregnancy, initiation of prenatal care, maternal age, maternal height, maternal weight before pregnancy, mother’s years of education, mother’s occupation during pregnancy, and total family income. From previous literature [8,27,38] these variables were known to have a significant relationship with neonatal birth weight and may increase the risk of having LBW neonate.

Based on the multiple regression analysis, as the reported average number of SHS exposure hours per week from home, work, and outside in the second trimester increased, the neonatal birth weight significantly decreased while holding constant the gestational age of the neonate, mother’s weight before pregnancy and mother’s weight gain during pregnancy. These covariates were the ones that were retained in the model after running the stepwise multiple regression analysis. Overall, the model was significant (R^2^ = 0.38; *F* (6,293) = 30.13; p < 0.05) (Table 5).

The total neonatal birth weight variance that was explained by the significant independent variables and significant covariates was 38%. The reported average number of SHS exposure hours per week from home, work, and outside in the second trimester explained 22% of the neonatal birth weight variance. The gestational age of the neonate, mother’s weight before pregnancy, and mother’s weight gain during pregnancy explained the remaining 16% of the neonatal birth weight variance.

### 3.6. The Risk of Having Low Birth Weight Neonate as a Result of SHS Exposure

A logistic regression was performed to test the second hypothesis. The estimated coefficients were the actual measures of the changes in the log odds ratio (ratio of the probabilities) of having LBW as the average number of SHS exposure increased one hour per week in each trimester while holding all covariates constant.

Based on logistic regression analysis, women who reported a higher average number of SHS exposure hours per week from occupational exposure in the second trimester were at significantly greater risk for having a LBW neonate than women who reported a lower average number of hours of SHS exposure from occupational exposure after controlling for mother’s weight before pregnancy, mother’s height, mother’s weight gain during pregnancy, total family income, and gestational age of the neonate. The adjusted odds ratio was OR = 1.331 (95% CI 1.052–1.684) for one unit increase of SHS exposure from work in the second trimester (p < 0.05). Women who reported a higher average number of SHS exposure hours per week from home and outside in the third trimester were at significantly greater risk for having a LBW neonate than women who reported a lower average number of hours of SHS exposure after controlling for the same covariates. The adjusted odds ratios were OR = 1.075 (95% C1 1.029–1.124) for one unit increase of SHS exposure from home in the third trimester and OR = 1.154 (95% CI 1.055–1.262) for one unit increase of SHS exposure from outside in the third trimester (p < 0.05) (Table 6).

With regard to significant covariates, subjects who reported a higher average weight before pregnancy were at significantly lower risk of having a LBW neonate than subjects who reported a lower average weight before pregnancy (B = −0.068, p < 0.05). The adjusted odds ratio was OR = 0.934 (95% CI 0.881–0.992) for one unit increase of mother’s weight before pregnancy. Subjects who reported a higher average weight gain during pregnancy were at significantly lower risk of having a LBW neonate than those who reported a lower average weight gain during pregnancy (B = −0.156, p < 0.05). The adjusted odds ratio was OR = 0.856 (95% CI 0.739–0.991) for one unit increase of weight gain.

In summary, as the reported average number of SHS exposure hours per week from all places (home, work, and outside) in the second trimester increased, the neonatal birth weight significantly decreased while holding all covariates constant (R^2^ = 0.38; *F* (6,293) = 30.13; p < 0.05). The SHS exposure variables accounted for 22% of the birth weight variance. Also, subjects who reported higher average number of SHS exposure hours per week from occupational exposure in the second trimester and from home and outside in the third trimester were at significantly greater risk for having a LBW neonate than women who reported lower average number of SHS exposure hours (p < 0.05). The adjusted odds ratio were OR = 1.331 (95% CI 1.052–1.684) for work exposure, OR = 1.075 (95% C1 1.029–1.124) for home exposure, and OR = 1.154 (95% CI 1.055–1.262) for outside exposure.

## 4. Discussion

Based on the results of this study, there was an adverse effect of SHS exposure on birth outcomes. This influence started from the second trimester and lasted through the third trimester. The results of this study are consistent with the results of previous studies, in which the researchers found that maternal exposure to SHS was associated with increased levels of carbon monoxide, nicotine, and cotinine in the serum or urine of the mother and the neonates and amniotic fluid [35,48].

The study results are also consistent with the results of previous studies, in which the researchers found that SHS exposure significantly reduced neonate’s birth weight [27,34,37,49]. The second trimester fetal growth is most rapid [7], and therefore that could explain the increased influence of SHS exposure starting from the second trimester. However, in maternal smoking and birth outcomes studies, smoking after the fourth month appears to be particularly crucial in lowering birth weight [37]. Interestingly, this study supports a recent systematic review and meta analysis [37] in which 58 studies were included to determine the effects of SHS exposure on birth outcomes. They proved estimates of 33g or more of birth weight reduction and increase the risk of birth weight below 2500 g by 22%, which represents a higher effect than older studies. In our study we found that the impact of SHS exposure is higher than previous estimates by the US Surgeon General’s report on the health consequences of SHS exposure [1]. Our findings are strengthened by the fact that maternal smoking as a confounding factor does not exist at all due to the fact that most Jordanian pregnant women do not smoke.

The findings of this study add new support to the growing evidence showing adverse effects of SHS exposure on birth outcome. The quantity of SHS exposure was an important predictor of birth weight. As the quantity of SHS exposure increased, the birth weight of the neonate significantly decreased. All types of SHS exposure (home, work, and outside) significantly reduced birth weight. The last two trimesters were the times in which SHS exposure significantly reduced birth weight. The findings related to timing of exposure were of particular importance since it was not investigated in the previously reviewed studies.

The study results are consistent with the conclusions of the World Health Organization. The International Consultation on Environmental Tobacco Smoke and Child Health concluded that maternal smoking during pregnancy is a major cause of reduced birth weight, decreased lung function, and sudden infant death syndrome (SIDS). Exposure to SHS among nonsmoking pregnant women can also cause a decrease in birth weight [50]. However, most of the evidence for these conclusions were drawn from studies conducted in the Western and developed countries. In this study, the relationship between SHS exposure and birth outcome was examined in Jordan, which is one of the developing countries that has a high prevalence of smoking among males (50%).

Smoking is on the rise in developing countries. Environmental conditions such as overcrowding may make the health effects of SHS more pronounced [28]. However, only two studies investigated the adverse health effects of SHS exposure in developing countries. The results of this study are consistent with the results of these two studies that were conducted in China [51] and India [28]. The authors of both studies found a significant relationship between SHS exposure and adverse birth outcomes.

In summary, based on the results of this study, SHS exposure during pregnancy exerts significant adverse effects on birth outcome. These results are consistent with the studies that were conducted in the developed countries, in the developing countries, and with the recommendations and conclusions of the WHO. The findings of this study add new support to the growing evidence showing adverse effects of SHS exposure on birth outcome. The quantity, type, and timing of exposure were all significant predictors of birth weight.

### 4.1. Significant Covariates

In this study, mother’s weight before pregnancy, mother’s weight gain, and gestational age of the neonate were significant predictors of neonate’s birth weight and accounted for 16% of its variance. Furthermore, mother’s weight before pregnancy and weight gain during pregnancy significantly decreased the risk of having a LBW neonate. These results are consistent with the results of previous studies. Higher weight gain during pregnancy and higher maternal pre-pregnancy weight were found to significantly decrease the risk of having a LBW neonate [52]. Maternal weight before pregnancy, representing the maternal long-term nutritional status, was an important independent determinant of birth weight and accounted for 13% of its variance. The weight gain during pregnancy, representing the short-term nutritional status, explained 5.6% of the variance [29]. Moreover, gestational age significantly decreased the neonatal birth weight [40].

In summary, mother’s weight before pregnancy, mother’s weight gain, and gestational age of the neonate were significant predictors of neonate’s birth weight. Controlling for these covariates is important while examining the influence of SHS exposure on birth weight.

### 4.2 Practice Implications

Birth weight is inversely correlated with both neonatal and postnatal mortality. The increased risk is not limited to LBW infants. Infants with birth weights of 3,000–3,499 grams have infant mortality rates that are higher than those of infants weighing 3,500–3,999 grams at birth [53]. However, reductions in birth weight that lead to LBW are much more hazardous. Approximately two-thirds of all infant deaths in the United States occurred to infants born with LBW. Low birth weight infants are also at an increased risk of neurological problems, including cerebral palsy, seizure disorders, severe mental retardation, lower respiratory tract conditions, and general morbidity [21]. Thus, it is conceivable that the reduction in birth weight, either less than 3,500 grams or less than 2,500 grams, caused by SHS exposure may increase the risk for morbidity and mortality during the first year of life. Based on this, it is important to review the implications of these study results for public health practice.

### 4.3. Public Health Implications

The goal of reducing LBW incidence by at least one third between 2000 and 2010 is one of the major goals in ‘A World Fit for Children’ [54]. Physical environment plays an important role in determining the birth weight and the future health of the infant. More than 20 million infants worldwide, representing approximately 16% of all births, are born with LBW, 95.6% of these are born in developing countries. The level of LBW in developing countries (16.5%) is more than double the level in developed countries (7%) [54].

Since nearly 1.1 billion people who smoked cigarettes consumed a total of five billion cigarettes in 1995, it is reasonable to assume that SHS exposure is a prevalent and an important public health problem. This is particularly true for developing countries, where men smoke substantially more than women (49% *versus* 9%) [55]. Swift action to highlight the need for strong public policies to protect infants and children from exposure to tobacco smoke is essential [50]. This can be achieved by two complementary strategies: eliminating infants’ contact with tobacco smoke in utero and in childhood and reducing overall consumption of tobacco products. Effectively implementing these strategies requires combining educational programs and legislative interventions aimed particularly at eliminating tobacco use in settings frequented by infants and children [50].

The workplace is also important to consider because women of childbearing age are present in the workplace in greater numbers than ever before. For example, women’s participation in the formal labor force in Jordan grew from 15% in 1980 to 24% in 1999 [56]. Moreover, the workplace is one source of SHS exposure for pregnant women that can and should be minimized to reduce the risk of adverse pregnancy outcomes for working women [57]. The safety of workplaces needs strong public health policies to protect working women. The antismoking regulations should be applied in the workplace, and they can be achieved by consultation with international organizations such as the World Health Organization, especially among developing countries like Jordan.

In summary, SHS exposure is a prevalent and an important public health problem, especially in developing countries. Exposure to SHS plays an important role in determining the birth weight. Combining educational programs and legislative interventions aimed particularly at eliminating tobacco use is the best public health strategy to reduce SHS influence on birth weight. Moreover, application of antismoking regulations in the workplace is a key component of the required public health policies.

### 4.4. Limitations

Although the study sample has the limitation of only being able to be generalized to those women who give birth in the public hospital of Irbid, Jordan, it has the strength of being systematically selected from this population. In Jordan, 95% of the deliveries occur in hospitals. Although 65% of these deliveries occur in public hospitals, public hospitals do not represent the whole population, since 35 % of deliveries occur in private hospitals. Also the self-reported measures of exposure are likely to be imprecise and subject to recall bias.

## Figures and Tables

**Table 1.** Month when the mother had the first prenatal visit.

	Frequency	Percent
First month	118	39.3
Second month	75	25.0
Third month	45	15.0
Fourth month	37	12.3
Fifth month	14	4.70
Sixth month	8	2.70
Seventh month	2	0.70
Eight month	1	0.30
Total	300	100

**Table 2.** Descriptive statistics for types of SHS exposures among study participants (n = 300).

	Frequency	Percent
Exposure from all places	6	2.00
Exposure from home and work only	2	0.70
Exposure from home and outside only	165	55.00
Exposure from work and outside only	4	1.30
Exposure from work only	1	0.30
Exposure from home only	23	7.70
Exposure from outside only	91	30.30
No exposure	8	2.70

**Table 3.** Mean SHS exposure hours per week from home, work, and outside in the first, second, and third trimesters.

	Minimum # of SHS exposure hours/ week	Maximum # of SHS exposure hours/ week	Mean of SHS exposure hours in the first trimester	Mean of SHS exposure hours in the second trimester	Mean of SHS exposure hours in the third trimester
Home	2	35	13.09	13.20	13.10
Work	2	15	5.00	4.800	4.54
Outside	0.5	21	4.80	4.86	4.85

**Table 4.** Comparison between the groups of participants who had normal birth weight (NBW) neonates and who had LBW neonates in terms of potential covariates (n = 300).

	NBW (*n* = 272)		LBW (*n* = 28)		*t*-test for equality of means		
	*M*	*SD*	*M*	*SD*	*t*	*DF*	*P*
Mother’s weight before pregnancy	63.57	10.59	56.96	7.82	4.095	38.0	0.000
Mother’s age	28.13	5.47	27.00	5.96	1.027	298	0.305
Mother’s height	160.32	6.33	159.64	5.56	0.545	298	0.586
Weight gain during pregnancy	11.27	3.48	9.82	3.23	2.112	298	0.036
Number of mother’s pregnancies	3.53	2.19	3.21	2.77	0.715	298	0.475
Number of years of education	11.40	2.77	11.39	2.99	0.014	298	0.989
Total family income	197.65	96.08	225.00	171.02	−0.833	28.8	0.412
Gestational age of the neonate	39.40	1.18	38.74	1.22	2.823	298	0.005

*Note:* α = 0.05M: mean; SD: standard deviation; DF: degree of freedom; p: probability value.

**Table 5.** Multiple regression analysis summary for variables predicting neonate’s birth weight (n = 300).

	Unstandardized coefficients		Standardized coefficients		☼*p*
	♦*B*	*SE	◙*β*	*○t*	
Number of hours per week in which the mother was exposed to SHS from her husband or someone else at home in the second trimester.	−17.92	2.24	−0.38	−8.00	0.000
Number of hours per week in which the mother was exposed to SHS from outside in the second trimester.	−25.98	4.98	−0.25	−5.21	0.000
Gestational age of the neonate.	58.02	17.28	0.16	3.36	0.001
Mother’s weight before pregnancy.	6.85	2.03	0.16	3.37	0.001
Number of hours per week in which the mother was exposed to SHS from work in the second trimester.	−51.54	16.31	−0.14	−3.16	0.002
Weight gain during pregnancy.	16.55	5.87	0.13	2.82	0.005

*Note: R = 0.618, R*^2^= 0.38, F = 6.29, P < 0.05, adjusted *R*^2^ *=* 0.369

♦*B: raw regression coefficients for each independent variable;*

*SE: Standard error for the regression coefficients;

◙*β: Beta weights value, standardized regression coefficient;*

☼*p: probability value;*

○*t: test statistics value.*

**Table 6.** Logistic regression analysis summary for variables predicting LBW (n = 300).

	95% CI for OR				
	*B*	*P*	OR	*Lower	**upper
Mother’s weight before pregnancy	−0.068	0.026	0.935	0.881	0.992
Mother’s height	0.066	0.135	1.068	0.980	1.164
Weight gain during pregnancy	−0.156	0.037	0.856	0.739	0.991
Total family income	0.002	0.278	1.002	0.998	1.006
Gestational age of the neonate	−0.358	0.073	0.699	0.472	1.034
Number of hours per week in which the mother was exposed to ETS from work in the second trimester	0.286	0.017	1.331	1.052	1.684
Number of hours per week in which the mother was exposed to ETS from her husband or someone else at home in the third trimester	0.073	0.001	1.075	1.029	1.124
Number of hours per week in which the mother was exposed to ETS from outside in the third trimester	0.143	0.002	1.154	1.055	1.262

*Note*: Cox & Snell R square = 0.156,

Hosmer and Lemeshow test Chi-square = χ^2^ (8, *n =* 300) = 7.99, *p* = 0.434,

Hit rate = 91.3%. *B* = estimated regression coefficient, OR = *e*^stimated coefficient;^

CI: Confidnet interval; OR: Odds ratio;

*: Lower Bound;

** Upper Bound.
